# Enhanced Expression of Human Epididymis Protein 4 (HE4) Reflecting Pro-Inflammatory Status Is Regulated by CFTR in Cystic Fibrosis Bronchial Epithelial Cells

**DOI:** 10.3389/fphar.2021.592184

**Published:** 2021-05-14

**Authors:** Zsolt Bene, Zsolt Fejes, Tibor Gabor Szanto, Ferenc Fenyvesi, Judit Váradi, Luka A. Clarke, Gyorgy Panyi, Milan Macek, Margarida D. Amaral, István Balogh, Béla Nagy

**Affiliations:** ^1^ Department of Laboratory Medicine, Faculty of Medicine, University of Debrecen, Debrecen, Hungary; ^2^ Department of Pediatrics, Faculty of Medicine, University of Debrecen, Debrecen, Hungary; ^3^ Kálmán Laki Doctoral School of Biomedical and Clinical Sciences, Faculty of Medicine, University of Debrecen, Debrecen, Hungary; ^4^ Department of Biophysics and Cell Biology, Faculty of Medicine, University of Debrecen, Debrecen, Hungary; ^5^ Department of Pharmaceutical Technology, Faculty of Pharmacy, University of Debrecen, Debrecen, Hungary; ^6^ Faculty of Sciences, BioISI-Biosystems and Integrative Sciences Institute, University of Lisboa, Lisboa, Portugal; ^7^ Department of Biology and Medical Genetics, Charles University—2nd Faculty of Medicine and Motol University Hospital, Prague, Czech; ^8^ Division of Clinical Genetics, Department of Laboratory Medicine, Faculty of Medicine, University of Debrecen, Debrecen, Hungary

**Keywords:** cystic fibrosis, inflammation, bronchial epithelial cell, HE4, CFTR modulator

## Abstract

Decreased human epididymis protein 4 (HE4) plasma levels were reported in cystic fibrosis (CF) patients under CFTR potentiator *ivacaftor* therapy, which inversely correlated with lung function improvement. In this study, we investigated whether HE4 expression was affected via modulation of CFTR function in CF bronchial epithelial (CFBE) cells *in vitro*. HE4 protein levels were measured in the supernatants of CFBE 41o^−^ cells expressing F508del-CFTR or wild-type CFTR (wt-CFTR) after administration of *lumacaftor/ivacaftor* or *tezacaftor/ivacaftor*, while HE4 expression in CFBE 41o^−^ cells were also analyzed following application of adenylate cyclase activators Forskolin/IBMX or CFTR_inh172_. The effect of all of these compounds on CFTR function was monitored by the whole-cell patch-clamp technique. Induced HE4 expression was studied with interleukin-6 (IL-6) in F508del-CFTR CFBE 41o^−^ cells under TNF-α stimulation for 1 h up to 1 week in duration. In parallel, plasma HE4 was determined in CF subjects homozygous for *p.Phe508del-CFTR* mutation receiving *lumacaftor/ivacaftor* (Orkambi^®^) therapy. NF-κB-mediated signaling was observed via the nuclear translocation of p65 subunit by fluorescence microscopy together with the analysis of IL-6 expression by an immunoassay. In addition, HE4 expression was examined after NF-κB pathway inhibitor BAY 11-7082 treatment with or without CFTR modulators. CFTR modulators partially restored the activity of F508del-CFTR and reduced HE4 concentration was found in F508del-CFTR CFBE 41o^−^ cells that was close to what we observed in CFBE 41o^−^ cells with wt-CFTR. These data were in agreement with decreased plasma HE4 concentrations in CF patients treated with Orkambi^®^. Furthermore, CFTR inhibitor induced elevated HE4 levels, while CFTR activator Forskolin/IBMX downregulated HE4 in the cell cultures and these effects were more pronounced in the presence of CFTR modulators. Higher activation level of baseline and TNF-α stimulated NF-κB pathway was detected in F508del-CFTR vs. wt-CFTR CFBE 41o^−^ cells that was substantially reduced by CFTR modulators based on lower p65 nuclear positivity and IL-6 levels. Finally, HE4 expression was upregulated by TNF-α with elevated IL-6, and both protein levels were suppressed by combined administration of NF-κB pathway inhibitor and CFTR modulators in CFBE 41o^−^ cells. In conclusion, CFTR dysfunction contributes to abnormal HE4 expression via NF-κB in CF.

## Introduction

Cystic fibrosis (CF; MIM:219700) is a monogenic disorder that is caused by pathogenic variants in the *cystic fibrosis transmembrane conductance regulator (CFTR)* gene (MIM:602421). CFTR protein is a chloride/bicarbonate channel, which regulates fluid transport across the apical membrane at various epithelial surfaces comprising e.g., the sweat gland, lumen of the bronchial tree and pancreatic exocrine ducts (Rowe et al., 2005). More than 2,000 variants have been identified in the *CFTR* gene with the major *p. Phe508del-CFTR* allele accounting for approximately 80% of all CF-causing alleles. CFTR dysfunction leads to impaired ion transport across epithelial surfaces resulting in airway dehydration and thick mucus secretion associated with chronic respiratory bronchial inflammation/obstruction that is further compounded by chronic lung colonization with pathognomonic bacteria, such as *P. aeruginosa* (Rowe et al., 2005). Neutrophil infiltration with high intrapulmonary protease levels (e.g., neutrophil elastase) and excess of proinflammatory cytokines, such as interleukin 6 (IL-6) are associated with “hyperinflammation” in the CF lung leading to the progressive damage of the bronchial tree (Nixon et al., 1998; Cantin et al., 2015).

The human epididymis protein 4 (HE4) is encoded by the *WFDC2* gene (MIM:617,548). Its enhanced expression was first detected in the lower, chronically inflamed CF airways according to immunohistochemistry findings (Bingle et al., 2006). Moreover, *WFDC2* was reported to be among the upregulated genes in the native CF nasal epithelium (Clarke et al., 2013). Since then, elevated serum HE4 concentration was positively associated with the overall disease severity of CF and the degree of pulmonary dysfunction in unrelated patient cohorts (Nagy et al., 2016). Additionally, HE4 mRNA levels were significantly higher in CF vs. non-CF airway biopsy specimens (Nagy et al., 2016).

Recently, CF patients treated with CFTR potentiator *ivacaftor* (IVA) and carrying at least one Class III *CFTR* CF-causing mutation (*p.Gly551Asp*) had lower plasma HE4 concentrations, which also inversely correlated with the improvement of their spirometry parameters (Nagy et al., 2019). Based on these preliminary results, serum or plasma HE4 concentration represents a novel biomarker that may be of value for routine monitoring of CFTR modulating therapy in CF (Bene et al., 2020).

However, the mechanism of abnormal HE4 expression in CF lung epithelial cells has not been investigated as yet. The link between CFTR dysfunction and chronic airway inflammation has been analyzed by former *in vitro* studies (Vij et al., 2009; Hunter et al., 2010). These data indicated that wild-type CFTR (wt-CFTR) has inherent anti-inflammatory properties that suppress baseline and stimulated NF-κB mediated inflammatory signaling in bronchial epithelial cells, while in CF abnormal CFTR function contributes to generally increased inflammation via disrupted suppression of the NF-κB pathway (Vij et al., 2009; Hunter et al., 2010). Similarly, aberrant *CFTR* expression and thus CFTR function reduce the ability of myeloid cells to successfully resolve infection and inflammation (Bonfield et al., 2012).

Introduction of CFTR modulator therapy renders a highly effective therapeutic modality which directly targets the basic CFTR defect and thus substantially improves the overall clinical course of CF (De Boeck and Amaral, 2016). In terms of the effect of CFTR modulators on cellular level of inflammatory processes, *lumacaftor/ivacaftor* (Orkambi^®^, LUM/IVA) treatment restores CFTR dependent chloride efflux (Favia et al., 2020) and decreases IL-18 and tumor necrosis factor α (TNF-α) expression in peripheral blood mononuclear cells (PBMCs) when measured in patients homozygous for the *p. Phe508del-CFTR* allele (Jarosz-Griffiths et al., 2020). This treatment with LUM/IVA also enhanced airway epithelial repair and thus improved transepithelial resistance, irrespective of the presence of *P. aeruginosa* (Adam et al., 2018). Similarly, *tezacaftor/ivacaftor* (Symdeko^®^, TEZ/IVA) downregulates serum IL-1β level at 3 months following its patient administration (Jarosz-Griffiths et al., 2020). Finally, in our recent clinical study, treatment with *ivacaftor* resulted in significantly lower plasma HE4 concentrations in three independent cohorts of CF patients already at 1 month following initiation of therapy (Nagy et al., 2019).

Currently, it is not clear whether HE4 expression is “directly” regulated by CFTR and thus could be influenced by CFTR modulators *in vitro* using human CF bronchial epithelial (CFBE) cells as a model cell culture system. Therefore, the major aims of this study are i) to determine HE4 level in the supernatants of cystic fibrosis bronchial epithelial (CFBE) 410^−^ cells expressing F508del-CFTR or wt-CFTR after *in vitro* administration of clinically relevant concentrations of CFTR modulators as well as CFTR activators and inhibitor; ii) to analyze HE4 concentrations in plasma samples drawn from CF subjects receiving Orkambi^®^; and iii) to investigate the role of NF-κB pathway in HE4 expression in association with impaired CFTR function and pro-inflammatory signaling in CF. To the best of our knowledge, such comprehensive approaches have not been applied thus far.

## Materials and Methods

### Reagents

CFTR correctors *lumacaftor* (VX-809, LUM) (S1565) and *tezacaftor* (VX-661, TEZ) (S7059)*,* CFTR potentiator *ivacaftor* (VX-770, IVA) (S1144), voltage-independent selective CFTR inhibitor CFTR_inh172_ (S7139), CFTR activator Forskolin (FSK, S2449), and NF-κB pathway inhibitor BAY 11-7082 (S2913) were purchased from Selleck Chemicals (Houston, TX, United States). cAMP phosphodiesterase inhibitor IBMX (3-isobuthyl-1-methylxanthine, I5879) was ordered from Sigma-Aldrich (St. Louis, MO, United States). Except for recombinant TNF-α (Gibco, Carlsbad, CA, United States), all reagents were dissolved in dimethyl sulfoxide (DMSO, Sigma-Aldrich).

### Cell Culture

CFBE 41o^−^ cells cultures stably expressing F508del-CFTR or wt-CFTR were grown in Minimum Essential Medium Eagle (EMEM) with Earle's BSS (EBSS) and 1% L-glutamine (Lonza, Walkersville, MD, United States), 10% fetal bovine serum (FBS, Sigma-Aldrich) and 5 μg/ml Puromycin (Sigma-Aldrich) at 37°C, 5% CO_2_ (Nagy et al., 2016). These cells were obtained from Dr J. P. Clancy’s lab (Cincinnati Children’s Hospital Medical Center, OH, United States). CFBE cells were seeded in 6-well plates (250.000 cells per well/sample). Supernatants for the analysis of HE4 and IL-6 protein levels were collected after CFBE cells were treated with TNF-α or phosphate buffer solution PBS, (i.e. at baseline) and combined CFTR modulators: corrector *lumacaftor* (3 μM) with potentiator *ivacaftor* (10 μM) (LUM/IVA) or corrector *tezacaftor* (5 μM) with *ivacaftor* (10 μM) (TEZ/IVA) or DMSO vehicle alone (thus representing the baseline) were administered for 24 h. For the activation of CFTR function, FSK (10 μM) with IBMX (100 μM) (FSK/IBMX) were added to both types of CFBE cells, while CFTR inhibition was carried out by CFTR_inh172_ (20 μM) in wt-CFTR CFBE cells vs. control samples with DMSO with or without CFTR modulators for 24 h. CFTR modulators were applied under similar experimental conditions as in comparable *in vitro* studies (Hunter et al., 2010; Wang et al., 2016; Pranke et al., 2017; Kmit et al., 2019). To investigate the role of NF-κB pathway in HE4 expression *in vitro*, BAY 11-7082 (5 μM) or DMSO (baseline) was used for 24 h to inhibit pro-inflammatory signaling in both unstimulated and TNF-α activated CFBE cells both in the presence or absence of LUM/IVA or TEZ/IVA molecules.

### Electrophysiology

Cl^−^ currents in CFBE 41o^−^ cells were measured in the whole-cell patch-clamp configuration similar to former publications (Boinot et al., 2014; Billet et al., 2017). The external (bath) solution contained 145 mM NaCl, 4 mM CsCl, 1 mM CaCl_2_, 1 mM MgCl_2_, 5 mM d-glucose, and 10 mM HEPES (pH 7.4 titrated with NaOH, 315 mOsm). The intracellular (pipette) solution contained 113 mM l-aspartic acid, 113 mM CsOH, 27 mM CsCl, 1 mM NaCl, 1 mM MgCl_2_, 1 mM ethylene glycol tetraacetic acid (EGTA), 10 mM HEPES, and 3 mM Mg-ATP (pH 7.2 titrated with CsOH, 285 mOsm). Mg-ATP was freshly diluted into the intracellular solution every hour. The intracellular solution was stored on ice before usage. FSK/IBMX and CFTR_inh-172_ were freshly diluted into the extracellular solution before the start of the experiments, respectively. Micropipettes were pulled in four stages by using a Flaming Brown automatic pipette puller (Sutter Instruments, San Rafael, CA, United States) from Borosilicate Standard Wall with Filament aluminum-silicate glass (GC150-TF10, Harvard Apparatus Co., Holliston, MA, United States) with tip diameters between 0.5 and 1 μm and heat polished to a tip resistance ranging typically 3–10 MΩ in the bath solution. All measurements were carried out by using Axopatch 200B amplifier connected to a personal computer using Axon Digidata 1,550 A data acquisition hardware, respectively (Molecular Devices Inc., Sunnyvale, CA, United States). The holding potential was maintained at −40 mV throughout the experiments, and two voltage-clamp protocols were used to measure whole-cell CFTR currents. First, a single depolarization from −40 to 0 mV was applied every 5 s for 4–5 min to monitor the current evolution and to confirm the absence of significant leak current. For determining the current–voltage (I–V) relationship the cells were held at a holding potential of −40 mV and depolarized to test potentials between −80 and +80 mV in steps of 20 mV increments every 10 s. Experiments were done at room temperature (RT) ranging between 20 and 24°C. Data were analyzed using the pClamp10.5 software package (Molecular Devices Inc.). Before analysis, current traces were digitally filtered with a three-point boxcar smoothing filter. Prior to analysis, current traces were corrected for ohmic leak.

### Total mRNA Extraction

Total mRNA from CFBE cell culture samples was isolated by TRI reagent (Molecular Research Center Inc., Cincinnati, OH, United States) according to the manufacturer’s recommendations. The purity and the concentration of separated mRNA samples were verified by a NanoDrop 2000 spectrophotometer (Thermo Scientific, Wilmington, DE, United States). Extracted mRNA samples were stored at −80°C before further analysis.

### Real-Time Quantitative PCR Analysis

Complementary DNA (cDNA) synthesis was performed with High Capacity cDNA Reverse Transcription kit (Applied Biosystems, Vilnius, Lithuania) according to the manufacturer’s protocol on extracted mRNA samples. The initial amount of RNA was 1,000 ng per reaction. Real-time quantitative PCR (RT-qPCR) was performed on a LightCycler 480 qPCR instrument (Roche Diagnostics, Mannheim, Germany) with LightCycler 480 SYBR Green I Master mix (Roche Diagnostics) including *WFDC2*-specific oligonucleotide primers (10 μM, Integrated DNA Technologies, Leuven, Belgium). The reactions were incubated at 95°C for 10 min, followed by 40 cycles of 95°C for 10 s and 60°C for 1 min. All measurements were run in triplicate. For normalization, we used the reference gene *RPLP0 (36B4)*. HE4 expression in TNF-α stimulated CFBE cells was monitored from 1 h up to 1 week vs. the baseline (using PBS) via measuring mRNA concentrations, while induced cellular activation was followed by IL6-, IL8- and IL1B-specific mRNA levels. Sequences of the primers for respective cDNA amplification are listed in Supplementary Table S1.

### Immunofluorescence Staining and Fluorescent Microscopy

Detection of the NF-κB pathway activation in CFBE 41o^−^ cells with or without CFTR dysfunction was initially visualized via p65 nuclear immunofluorescence staining based on the method drawn from our previous study (Fejes et al., 2018) with some modifications. For this purpose, F508del-CFTR and wt-CFTR CFBE cells were cultured in 12-well plates on sterile glass microscope slides at a density of 5 × 10^4^ cells/slide for 2 days. Cells were then treated with TNF-α (100 ng/mL) or vehicle (PBS, baseline) for 4 h. When the impact of CFTR modulator treatment on activation level of NF-κB pathway was studied, CFBE cells were preincubated with *lumacaftor* (3 μM) with *ivacaftor* (10 μM) or *tezacaftor* (5 μM) with *ivacaftor* (10 μM) or DMSO (baseline) for 24 h, and with TNF-α (100 ng/ml) or PBS (baseline) was added for 4 h. After these pretreatments, cells were fixed with ice-cold methanol-acetone (50 v/v %) for 10 min. Non-specific antibody binding sites were blocked with fetal bovine serum (FBS, Sigma-Aldrich) for 15 min. For primary labeling of NF-κB p65 subunit, polyclonal rabbit anti-human p65 antibody (100 μg/ml, Sigma-Aldrich) was used for 1 h followed by secondary staining with Alexa Fluor 488-conjugated goat-anti-rabbit IgG (5 μg/ml, Sigma-Aldrich) for 1 h. Cell nuclei were labeled with 4′,6-diamidino-2-phenylindole (DAPI, Invitrogen, Carlsbad, CA, United States), and samples were observed by Zeiss Axio Scope. A1 fluorescent microscope (HBO 100 lamp) (Carl Zeiss Microimaging GmbH, Goettingen, Germany). DAPI: excitation at 365 nm, emission BP filter 445/50 nm; fluorescein: excitation of BP filter at 470/40 nm, emission BP filter 525/50 nm. Images were analyzed with ZEN 2012 v.1.1.0.0. software (Carl Zeiss Microimaging GmbH). The ratio of nuclear and perinuclear (cytosol) fluorescence intensity was calculated for NF-κB p65 staining. The specificity of immunostaining was checked by incubating the cells with the secondary antibody only, and where very limited background staining was seen.

### CF Patients

Ten CF patients with the classical and stable form of the disease and being homozygous for the *p. Phe508del-CFTR* pathogenic variant (5 females and 5 males, mean age of 16.1 ± 4.8 years) were randomly selected from a pre-existing cohort in order to measure plasma HE4 levels in samples obtained at 1 month of Orkambi^®^ administration (Vertex Pharmaceuticals, Boston, MA, United States) (Supplementary Table S2). These subjects formerly participated in the PROSPECT study (ClinicalTrials.gov identifier: NCT0247731), and samples were requested from Cystic Fibrosis Foundation Therapeutics (CFFT) Biorepository (Bethesda, MD, United States). Mean value of baseline forced expiratory volume in 1 s (FEV_1_% predicted) of these subjects was 74.6 ± 16.6%. Before treatment, mean sweat chloride concentration was 101.2 ± 9.2 mmol/L, while the mean change of sweat chloride was −19.6 mmol/L. Aliquots of their blood samples were obtained through venous puncture, and were centrifuged, then stored at −80°C. K_3_-EDTA anticoagulated plasma aliquots were transferred from CFFT for HE4 analysis by international courier service on dry ice to the Department of Laboratory Medicine, University of Debrecen, Hungary.

### Laboratory Analyses

Chemiluminescent microparticle immunoassay (Architect-i1000SR^®^, Abbott Diagnostics, Wiesbaden, Germany) was used to analyze protein levels of HE4 in the supernatants obtained from CFBE cell cultures following different research treatments as indicated above. In addition, HE4 plasma concentrations were measured before treatment and after LUM/IVA treatment with the same immunoassay that we used on our previous cohorts (Nagy et al., 2019). IL-6 levels were measured by electro-chemiluminescent immunoassay on a Cobas e 411 instrument (Roche Diagnostics). These measurements were performed in an analyst-blinded mode in all studied cases in order to avoid any potential operator-related bias.

### Ethics Statement

This study was approved by the Regional Ethics Committee of the University of Debrecen (permit number: 4813-2017) in accordance with the World Medical Association Declaration of Helsinki—Ethical Principles for Medical Research Involving Human Subjects.

### Statistical Analysis

The Kolmogorov-Smirnov test was used for the evaluation of the normality of the data. Data are expressed in mean ± SD or SEM, where applicable. Unpaired *t*-test or Mann-Whitney *U* test was performed to compare two groups of data, while comparison of multiple groups was performed using the ANOVA with Bonferroni’s multiple comparisons test. For comparison of plasma HE4 levels before and under CFTR modulator treatment, paired *t*-test was utilized. The *p* < 0.05 probability level was regarded as being statistically significant. Analyses were performed using GraphPad Prism, version 6.01 (GraphPad Software, La Jolla, CA, United States).

## Results

### CFTR Modulators Partially Rescue F508del-CFTR Cl^−^ Currents in CFBE 41o^−^ cells

**FIGURE 1:**
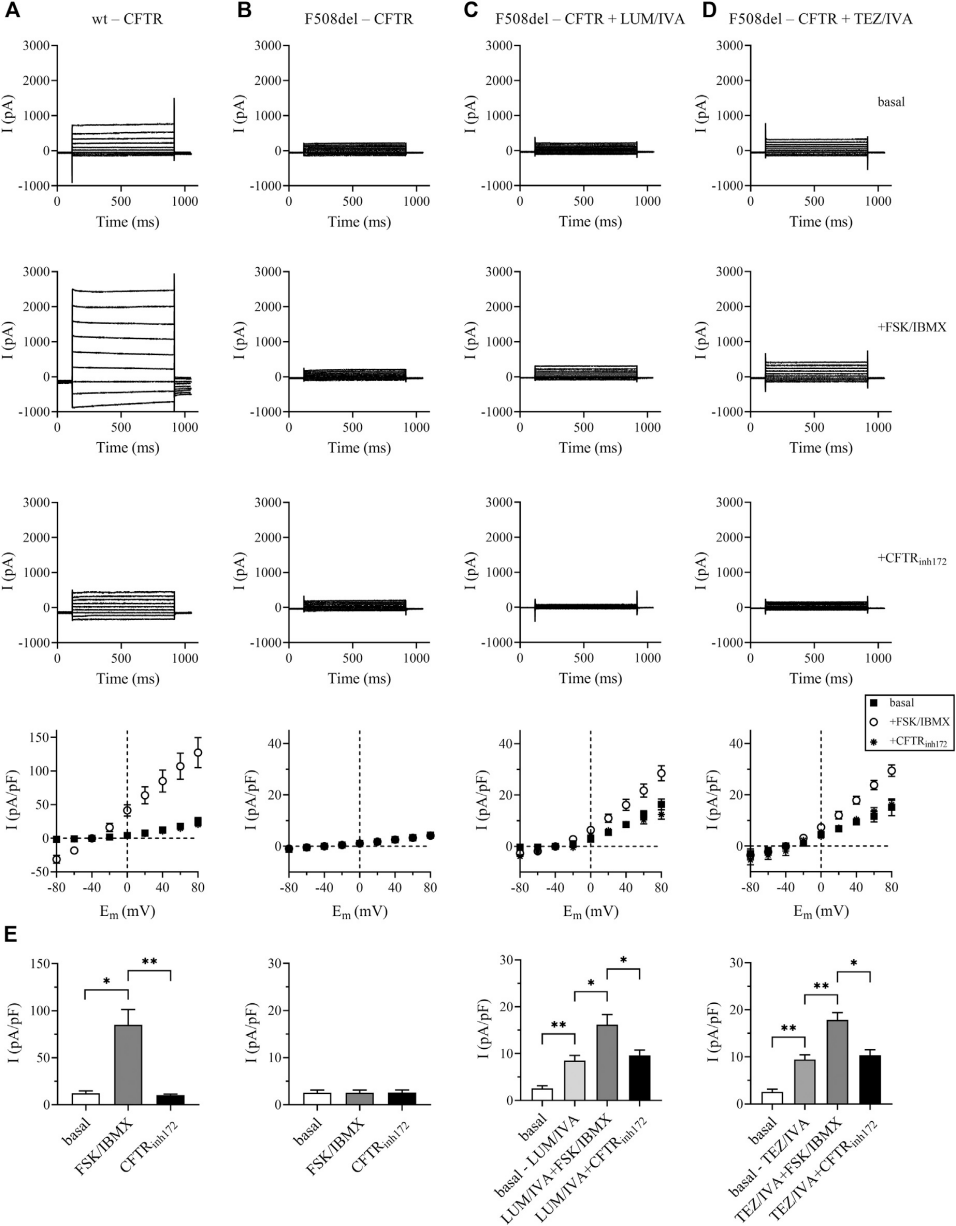
Rescue of functional F508del-CFTR Cl^−^ currents in LUM/IVA and TEZ/IVA-treated CFBE 41o^−^ cells. Representative traces of whole-cell Cl^−^ currents were elicited by stepping from a holding potential of −40 mV to series test potentials ranging from −80 to +80 mV with 20-mV increments every 10 s in CFBE 41o^−^ cells expressing the wild-type CFTR [wt-CFTR, column **(A)**], the F508del-CFTR [F508del-CFTR, column **(B)**], or in cells expressing the deletion mutant CFTR but treated for 24 h with LUM/IVA [3/10 μM, column **(C)**], or TEZ/IVA [5/10 μM, column **(D)**]. Currents were measured at RT. Top traces: basal current, in the absence of FSK/IBMX stimulation; middle traces: after ∼2 min stimulation by FSK/IBMX (10/100 μM); bottom traces: in the presence of FSK/IBMX and 20 μM of the CFTR inhibitor CFTR_inh172_. The bottom panels show the peak current density-voltage relationships (pA/pF, mean ± SEM, n = 3–5) in the absence of FSK/IBMX (basal, filled squares), upon stimulation by FSK/IBMX (open circles) and in the presence of FSK/IBMX and 20 μM CFTR_inh172_ (asterisks). Histograms of the corresponding current densities (pA/pF) determined at +40 mV. Data are expressed in mean ± SEM, n = 3–5 cells/condition **(E)** Basal: wild-type CFTR expressing cells (left panel) or F508del-CFTR expressing cells (all other panels) in the absence of FSK/IBMX stimulation. Basal-LUM/IVA and basal-TEZ/IVA: F508del-CFTR expressing cells treated with the CFTR modulators only (see above), FSK/IBMX: stimulation of basal or LUM/IVA or TEZ/IVA-treated cells by FSK/IBMX (see above); CFTR_inh172_: basal or LUM/IVA or TEZ/IVA-treated cells in the presence of FSK/IBMX and CFTR_inh172_. Unpaired or paired *t*-test was performed for comparisons. ^*^ *p* < 0.05, ^**^ *p* < 0.01.

In order to demonstrate that the CFTR modulators applied in this study restore CFTR function in airway epithelial cells *in vitro,* we treated human CFBE 410-cell cultures expressing F508del-CFTR with two different combinations of CFTR modulators (LUM/IVA or TEZ/IVA) for 24 h and analyzed Cl^−^ current density using patch-clamp. CFBE 41o^−^ cells expressing wild-type CFTR showed whole-cell Cl^−^ currents that could be robustly activated by FSK/IBMX and inhibited by the CFTR selective inhibitor CFTR_inh172_. The peak current-voltage relationship indicated (Figure 1A, bottom panel) a linear current-voltage relationship, which was most obvious after FSK/IBMX treatment, that reversed around −40 mV, the expected reversal potential of a Cl^−^ current calculated from the ionic composition of the pipette-filling and extracellular solutions. The same experiments in CFBE 41o^−^ cells expressing F508del-CFTR resulted in miniature currents that were insensitive to either FSK/IBMX activation or inhibition by CFTR_inh172_ (Figure 1B). Most importantly, both combinations of CFTR modulators (LUM/IVA, Figure 1C, or TEZ/IVA, Figure 1D) significantly increased the basal and the FSK/IBMX-stimulated Cl^−^ current in comparison to F508del-CFTR basal Cl^−^ current (Figures 1B–D). Moreover, the currents activated by FSK/IBMX treatment were sensitive to CFTR_inh172_ (Figures 1C,D). The statistical analysis of the current densities at +40 mV in Figure 1E confirms that CFTR modulators corrected F508del-CFTR channel function (Figure 1E). The current densities recorded in the presence of FSK/IBMX in cells treated with either LUM/IVA or TEZ/IVA were comparable to the wt-CFTR current density in the absence of the activators (∼20 pA/pF) and smaller than wt-CFTR current after stimulation (∼80 pA/pF). Of note, TEZ/IVA restored F508del-CFTR Cl^−^ current density at a moderately higher level than LUM/IVA (9.44 ± 1.01 vs. 8.48 ± 1.14 pA/pF; p = 0.560). In summary, LUM/IVA and TEZ/IVA CFTR modulators partially restored CFTR function in CFBE 41o^−^ cells cultures expressing F508del-CFTR.

### CFTR Function Modulates HE4 Concentrations in CFBE 41o^−^ cells Culture Supernatants *in vitro*

**FIGURE 2:**
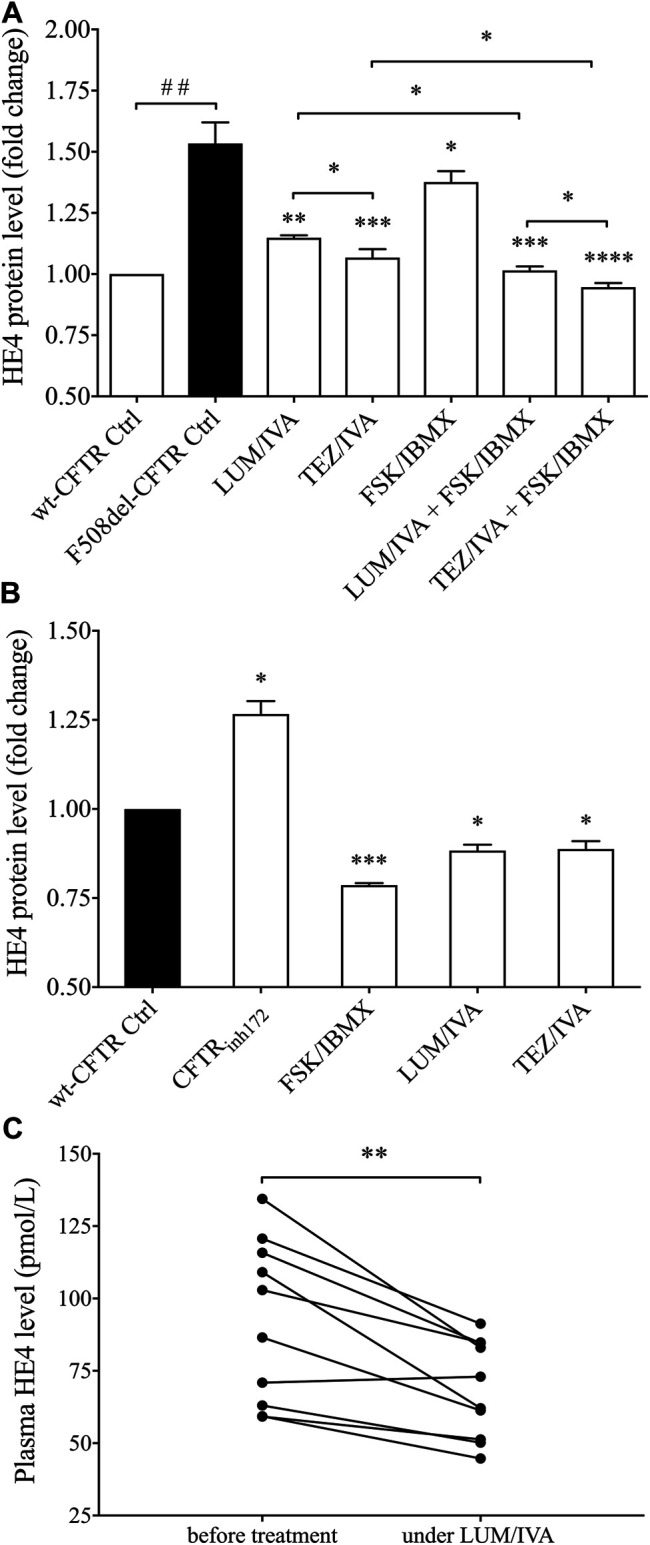
Analysis of HE4 levels in the supernatant of F508del-CFTR or wt-CFTR CFBE 41o^−^ cells with pharmacologically altered CFTR function and in plasma samples of CF subjects under LUM/IVA therapy. Both LUM/IVA (3/10 μM) and TEZ/IVA (5/10 μM) resulted in decreased HE4 in F508del-CFTR CFBE 41o^−^ cells after 24 h approaching the level of normal cells. CFTR activator FSK/IBMX (10/100 μM) alone moderately reduced HE4, while combined treatment of FSK/IBMX with CFTR modulators showed an even larger change in HE4 **(A)** CFTR_inh172_ (20 μM) caused elevated HE4 levels after 24 h while, in turn, improved CFTR function with FSK/IBMX or CFTR modulator reduced HE4 even in wt-CFTR CFBE 41o^−^ cells **(B)** There were lower plasma HE4 values compared to baseline measured in CF subjects (n = 10) after 1 month of Orkambi^®^ in agreement with *in vitro* data with LUM/IVA **(C)** Data are expressed in mean ± SEM (n = 5–6 samples/condition). Unpaired or paired *t*-test or Mann-Whitney *U* test was performed for comparisons. ^##^ *p* < 0.01 vs. wt-CFTR cells; ^*^ *p* < 0.05, ^**^ *p* < 0.01, ^***^ *p* < 0.001, and ^****^ *p* < 0.0001 vs. F508del-CFTR control (ctrl) cells with DMSO, or baseline plasma samples, respectively.

CFBE 410-cell cultures expressing F508del-CFTR were treated with CFTR modulators LUM/IVA or TEZ/IVA for 24 h to quantify protein levels of HE4 in the cell culture supernatants. Subsequently, CFBE 41o^−^ cells with wt-CFTR were utilized as controls for HE4 supernatant concentrations. We consistently found that baseline HE4 concentration was higher in F508del-CFTR CFBE 41o^−^ cells than normal cells (*p* < 0.01) and was significantly reduced by LUM/IVA (*p* < 0.01) and TEZ/IVA (*p* < 0.001) treatment compared to control samples where the vehicle (i.e., DMSO) was applied. The concentration of HE4 in the supernatant was close to that observed in wt-CFTR CFBE 41o^−^ cells (Figure 2A). Interestingly, TEZ/IVA caused a larger decrease of HE4 concentrations compared to LUM/IVA (*p* < 0.05). In parallel, CFTR activator FSK/IBMX was used alone and in combination with aforementioned CFTR modulators in cells with F508del-CFTR. The HE4 protein level was decreased by FSK/IBMX (*p* < 0.05) likely due to the activation of residual CFTR function, while further reduction in HE4 was observed after the combined treatment (*p* < 0.05) vs. using individual CFTR modulator molecules (Figure 2A).

To provide further evidence of the inverse association between HE4 expression measured by its concentration in cell culture supernatants and CFTR function, we also applied pharmacological inhibition of CFTR by CFTR_inh172_ in wt-CFTR CFBE 41o^−^ cells, which caused elevated HE4 levels (*p* < 0.05). In contrast, there was a significant decrease in HE4 concentrations (*p* < 0.001) after FSK/IBMX treatment when compared to the controls. Interestingly, LUM/IVA and TEZ/IVA could downregulate HE4 expression to a certain extent even in CFBE 41o^−^ cells with normal CFTR expression (*p* < 0.05) (Figure 2B). These data suggest that CFTR function affects basal levels of HE4 expression and impaired function of CFTR could explain elevated HE4 concentration in CF airway epithelial cells *in vitro*.

### Treatment With LUM/IVA Lowers Plasma HE4 Levels in CF Subjects Homozygous for *p.Phe508del-CFTR* Mutation

To substantiate *in vivo* our *in vitro* results above, we determined plasma HE4 levels in 10 randomly selected CF individuals homozygous for *p. Phe508del-CFTR* mutation who were under Orkambi^®^ (LUM/IVA) medication. In the presence of decreasing sweat chloride concentrations (Supplementary Table S2), there were significantly (*p* < 0.01) reduced HE4 plasma concentrations—regardless of its baseline value—already at 1 month of treatment, this being the earliest follow-up time point of these patients (Figure 2C). These clinical data underscore the impact of CFTR modulation therapy on decreasing HE4 plasma concentrations in CF and are in agreement with previous findings in IVA monotherapy in cases with at least one *p. Gly551Asp-CFTR* pathogenic variant (Nagy et al., 2019).

### TNF-α Induces Increased *HE4* mRNA Expression in CFBE 41o^−^ cells Cultures *in vitro*

**FIGURE 3:**
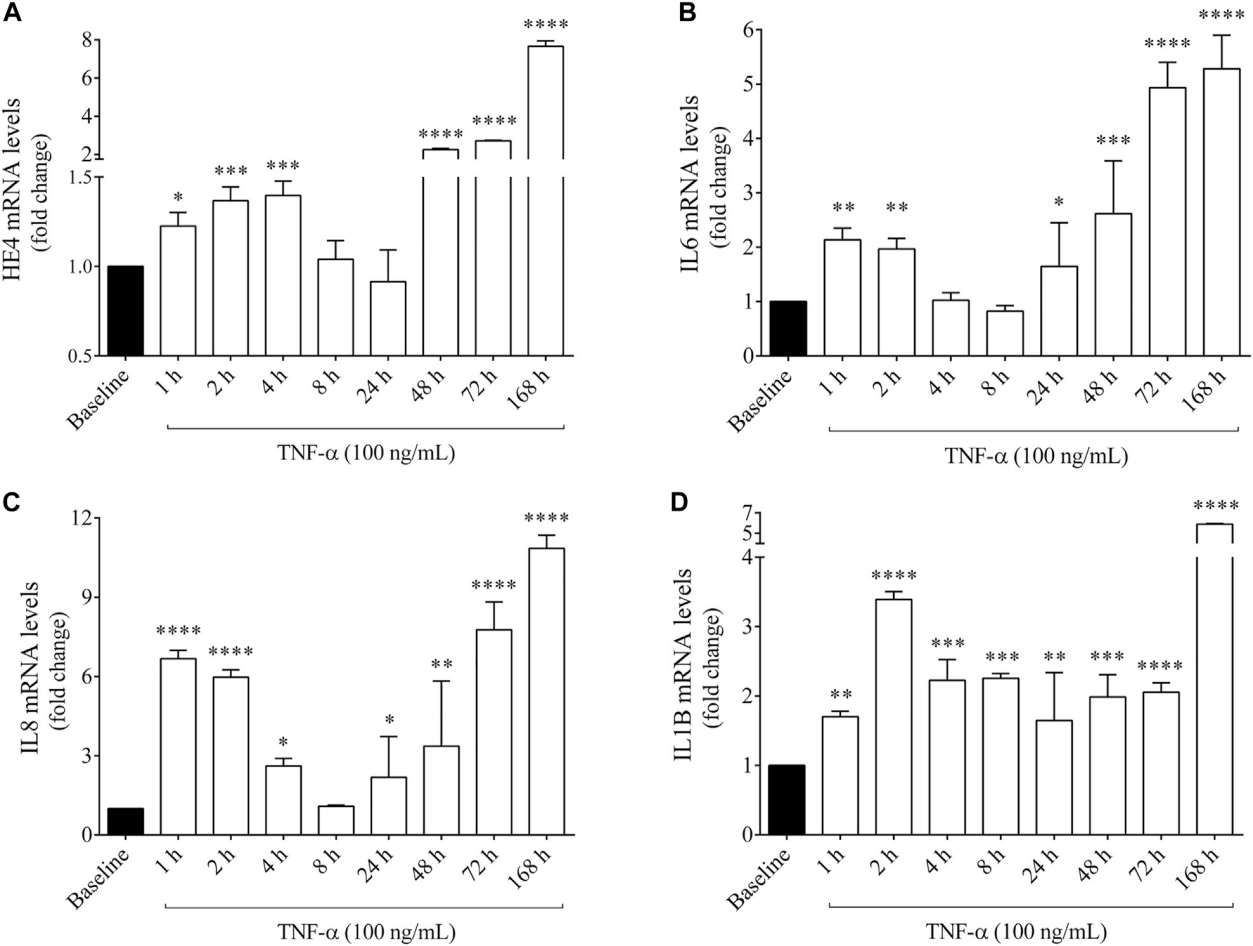
Quantification of HE4, IL6, IL8 and IL1B mRNA levels in F508del-CFTR CFBE 41o^−^ cells after TNF-α stimulation for 1 h up to 1 week *in vitro*. HE4 mRNA level was higher than baseline at 1–4 h that returned to normal range by 24 h and was further induced by TNF-α (100 ng/ml) after 48 h up to 168 h **(A)** IL6 mRNA level was already induced by 1–2 h and was further augmented after 24 h **(B)** In parallel, IL-8 expression showed a similar time-course alteration to HE4 and IL6 **(C)**, while IL1B mRNA level had a sustained elevation without a substantial alteration during this time period **(D)** Mean ± SEM (n = 4–5 samples/condition). ^*^ *p* < 0.05, ^**^ *p* < 0.01, ^***^ *p* < 0.001, and ^****^ *p* < 0.0001 vs. baseline, based on ANOVA with Bonferroni’s multiple comparison test.

Subsequently, we studied whether *WFDC2/HE4* expression could be further enhanced by an artificial inflammatory stimulus *in vitro*. For this purpose, F508del-CFTR CFBE 41o^−^ cells were stimulated with recombinant TNF-α applied in the range spanning from 1 h up to 1 week. As a result, HE4 mRNA level quantified by RT-qPCR raised already after 1 h of treatment vs. untreated (baseline) sample (*p* < 0.05) and was further elevated within the period of 4 h (*p* < 0.001). Surprisingly, HE4 mRNA levels returned to baseline within 24 h. When TNF-α was administered for longer periods (from 48 h up to 1 week), there was a much higher expression of HE4 mRNA (*p* < 0.0001) in TNF-α stimulated CFBE 41o^−^ cells (Figure 3A).

In the same set of samples, mRNA levels of pro-inflammatory cytokines IL-6, IL-8 and IL-1β (Figures 3B–D) were also analyzed to ascertain if these mediators were also provoked by TNF-α together with increased expression of HE4. In this regard, IL-6 and IL-8 expression showed similar time-dependent alteration patterns as observed in HE4 mRNA, while elevated IL1B mRNA was sustained from 1 h without a substantial change throughout this time period. Furthermore, the protein concentrations of HE4 and IL-6 were measured in the supernatants of F508del-CFTR CFBE 41o^−^ cells at some selected time points (between 4 and 168 h), whereby HE4 level was significantly elevated after 4 h and gradually increased up to 1 week of treatment, while IL-6 concentration was significantly augmented at all pre-selected time points (Supplementary Figures S1A,B). These results imply that expression of HE4 mRNA thus resulting concentrations of HE4 protein are upregulated following TNF-α administration accompanied by different pro-inflammatory cytokines in CFBE cells.

### The Pro-inflammatory NF-κB Pathway is Influenced by the Combination of CFTR Modulators in F508del-CFTR CFBE 41o^−^ cells *in vitro*

**FIGURE 4:**
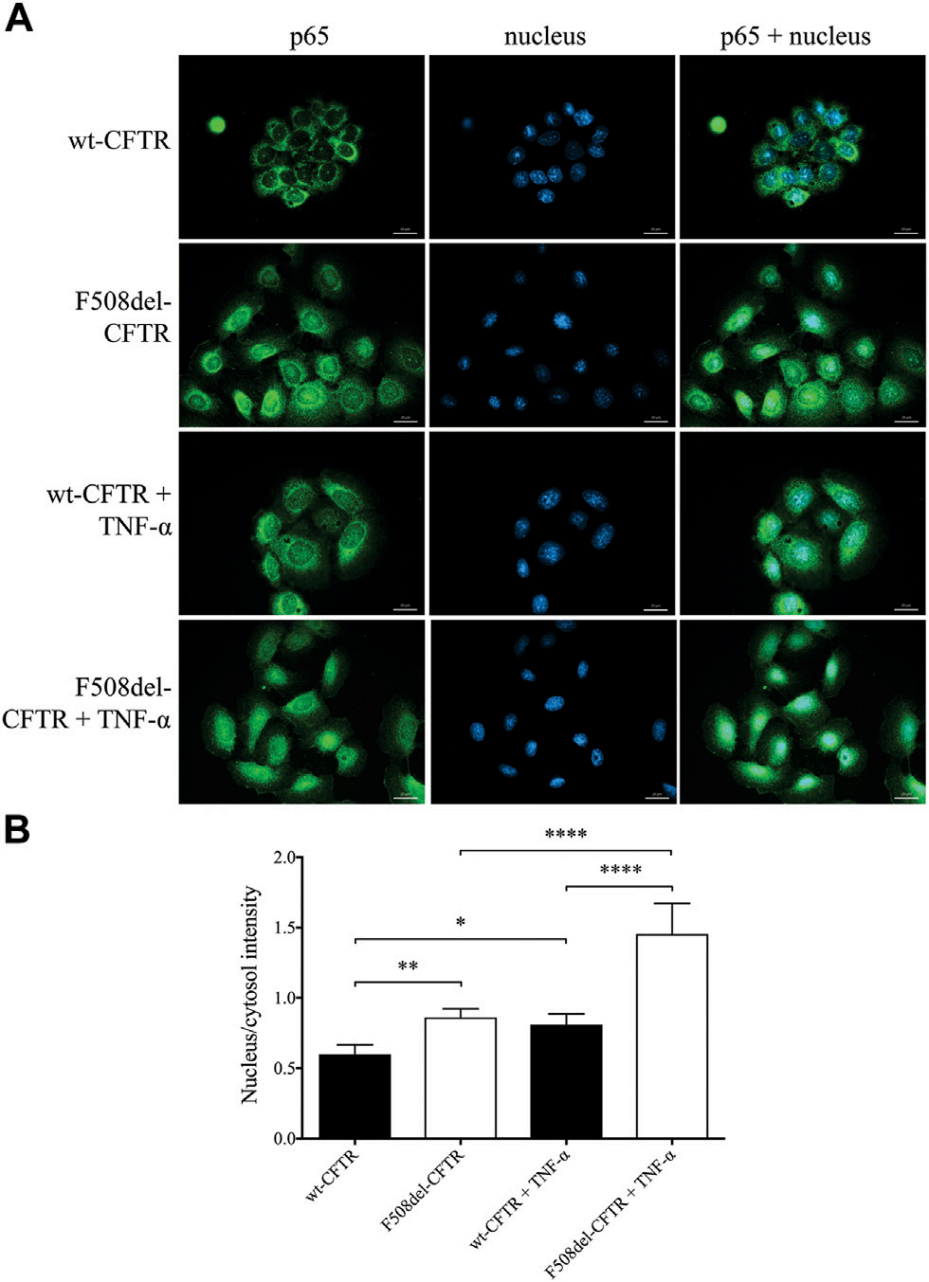
Immunofluorescence staining with the quantification of nuclear/cytosol p65 positivity for detecting NF-κB pathway activation in unstimulated and TNF-α treated CFBE 41o^−^ cells expressing F508del-CFTR or wt-CFTR. The basal level of p65 nuclear translocation was analyzed in respect to CFTR function when normal and F508del-CFTR CFBE 41o^−^ cells were stimulated with 100 ng/ml TNF-α or PBS (baseline) for 4 h. Green: p65 staining; blue: cell nuclei. Scale bar: 20 μm **(A)** Fluorescence intensity of the NF-κB immunostaining was analyzed based on the ratio of the nucleus/cytosol intensity **(B)** Mean ± SEM (n = 6–7 cells/condition). ^*^ *p* < 0.05, ^**^ *p* < 0.01 and ^****^ *p* < 0.0001 based on statistical analyses.

**FIGURE 5:**
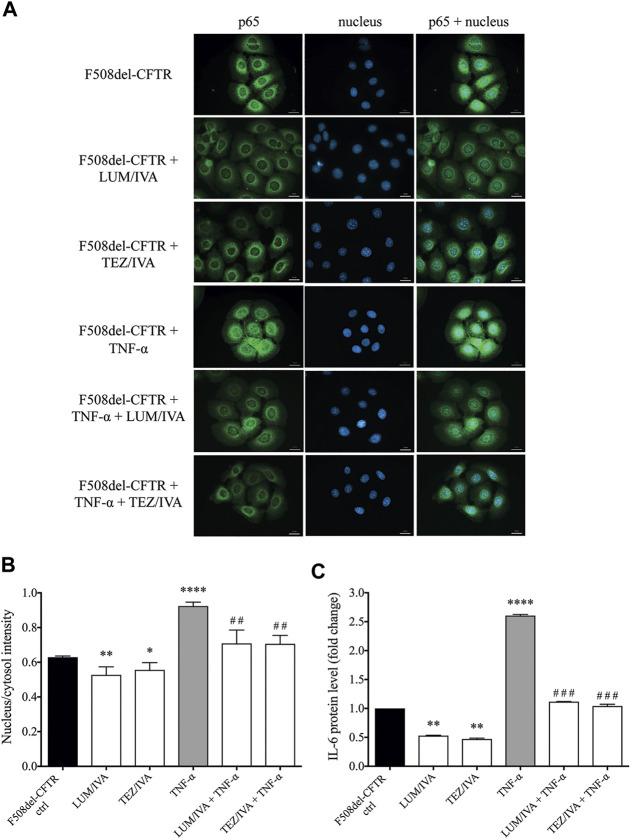
Immunofluorescence staining and quantification of p65 nuclear translocation staining with the analysis of IL-6 protein levels in the presence of CFTR modulators in unstimulated or TNF-α treated CFBE 41o^−^ cells cultures expressing F508del-CFTR. Alteration in p65 nuclear translocation was first analyzed when cells were pretreated with LUM/IVA or TEZ/IVA or DMSO (baseline) for 24 h. CFBE cells were then activated with 100 ng/ml TNF-α or PBS (baseline) for 4 h. Green: p65 staining; blue: cell nuclei. Scale bar: 20 μm **(A)** Fluorescence intensity of the NF-κB immunostaining was analyzed based on the ratio of the nucleus/cytosol intensity **(B)** Downregulated IL-6 protein levels were measured in the supernatants of untreated and TNF-α stimulated CFBE 410-cells to approve the inhibition of NF-κB pathway activation by CFTR modulators by 24 h **(C)** Mean ± SEM, n = 4–5 cells/condition. ^*^ *p* < 0.05, ^**^ *p* < 0.01, and ^****^ *p* < 0.0001 vs. control cells with DMSO; ^##^ *p* < 0.01 and ^###^ *p* < 0.001 vs. CFBE cells with TNF-α and DMSO.

To establish the relationship between upregulated NF-κB pathway due to CFTR dysfunction and the abnormal HE4 expression in CFBE cells *in vitro*, we applied two experimental approaches. First, the activity of NF-κB pathway was assessed via the p65 nuclear translocation experiments. This protein, also known as RelA (MIM:164,014), is one of the 5 components that constitute the NF-κB transcription factor family and is used as a marker of NF-κB pathway activation. We applied fluorescence microscopy-based approaches in wt-CFTR and F508del-CFTR CFBE 41o^−^ cells that had been treated with TNF-α vs. untreated controls. Second, the effect of CFTR modulators was studied *in vitro* on basal and induced NF-κB signaling in these CFBE cells after the application of LUM/IVA or TEZ/IVA treatment via p65 nuclear positivity and IL-6 in the cell line supernatants. There was a significantly higher baseline level of p65 positivity in the nuclei of F508del-CFTR CFBE 41o^−^ cells vs. normal cells (*p* < 0.01). When these cell cultures were exposed to TNF-α, significantly higher p65 positivity was seen in both cell types (*p* < 0.05, *p* < 0.0001, respectively), and the difference in p65 translocation between normal and deficient CFBE cells was more pronounced (*p* < 0.0001) (Figures 4A,B). These results indicate that there is a higher baseline and induced level of inflammatory status in CF vs. normal CFBE cells. Secondly, CFTR modulators substantially decreased p65 positivity (*p* < 0.05) not only in unstimulated F508del-CFTR CFBE 41o^−^ cells, but a significant reduction was also observed (*p* < 0.001) after TNF-α treatment compared to baseline (DMSO) sample (Figures 5A,B). In parallel, we determined IL-6 protein levels in the supernatants of studied CFBE cell lines which confirmed the anti-inflammatory effect of applied CFTR modulators via downregulation of basal and TNF-α stimulated IL-6 expression (Figure 5C). Hence, our data provide evidence that the generally increased levels of NF-κB pathway activation due to CFTR dysfunction could be efficaciously downregulated by the application of LUM/IVA or TEZ/IVA in CFBE cell cultures *in vitro*.

### HE4 Concentrations in CFBE 41o^−^ cells Cultures Are Not Only Upregulated by the NF-κB Pathway and Pro-Inflammatory Signaling but Are Also Directly Influenced by CFTR Activity

**FIGURE 6:**
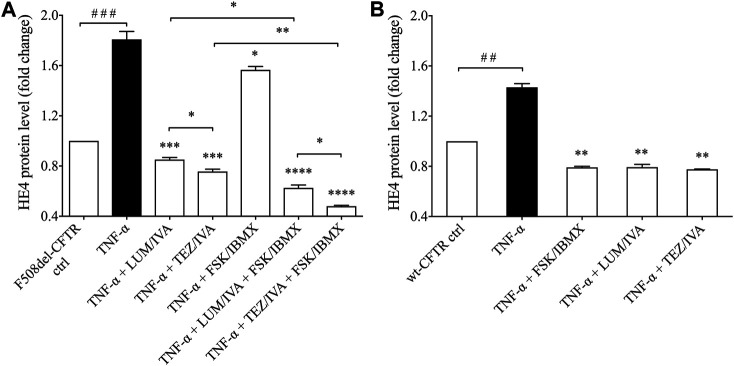
Determination of HE4 level in TNF-α activated F508del-CFTR or wt-CFTR CFBE 41o^−^ cells with *in vitro* modulated CFTR function. First, TNF-α activated F508del-CFTR CFBE 41o^−^ cells were analyzed under the treatment with LUM/IVA, TEZ/IVA, FSK/IBMX or their combination for 24 h. TNF-α could not elevate HE4 expression in the presence of improved CFTR function **(A)** In parallel, CFTR function was affected by FSK/IBMX or CFTR modulators in TNF-α-stimulated wt-CFTR CFBE 41o^−^ cells for 24 h. Similar to the results in F508del-CFTR CFBE 41o^−^ cells, HE4 expression remained lowered compared to samples with TNF-α but with a smaller extent **(B)** Mean ± SEM, n = 5–6 samples/condition. ^*^ *p* < 0.05, ^**^ *p* < 0.01, ^***^ *p* < 0.001 and ^****^ *p* < 0.0001 vs. control samples with TNF-α and DMSO; ^##^ *p* < 0.01 and ^###^ *p* < 0.001 vs. control samples with PBS.

Finally, we wanted to examine if TNF-α induced HE4 levels could also be decreased by applying CFTR modulators in F508del-CFTR CFBE 41o^−^ cells. Administration of either LUM/IVA or TEZ/IVA resulted in a significant reduction in HE4 concentration (*p* < 0.001) when measured in the supernatants of TNF-α activated CFBE 41o^−^ cells vs. controls with TNF-α and DMSO (Figure 6A). Furthermore, FSK/IBMX alone caused a moderate but still significantly reduced HE4 level in the presence of TNF-α (*p* < 0.05), whereas combined administration of LUM/IVA or TEZ/IVA with FSK/IBMX lowered HE4 concentrations by a significantly higher degree (*p* < 0.01; *p* < 0.001, respectively). Similar to the unstimulated samples (Figure 2A), TEZ/IVA caused a larger change in HE4 compared to LUM/IVA with or without CFTR activator (*p* < 0.05). (Figure 6A). When TNF-α activated wt-CFTR epithelial cells were treated with CFTR activator FSK/IBMX or CFTR modulators, there were similarly reduced HE4 levels (*p* < 0.01) compared to TNF-α activated samples with vehicle (Figure 6B). Thus, observed data provide evidence that corrected and/or potentiated CFTR function has a “protective role” against TNF-α-induced upregulation of HE4 expression in CFBE cells *in vitro*.

**FIGURE 7:**
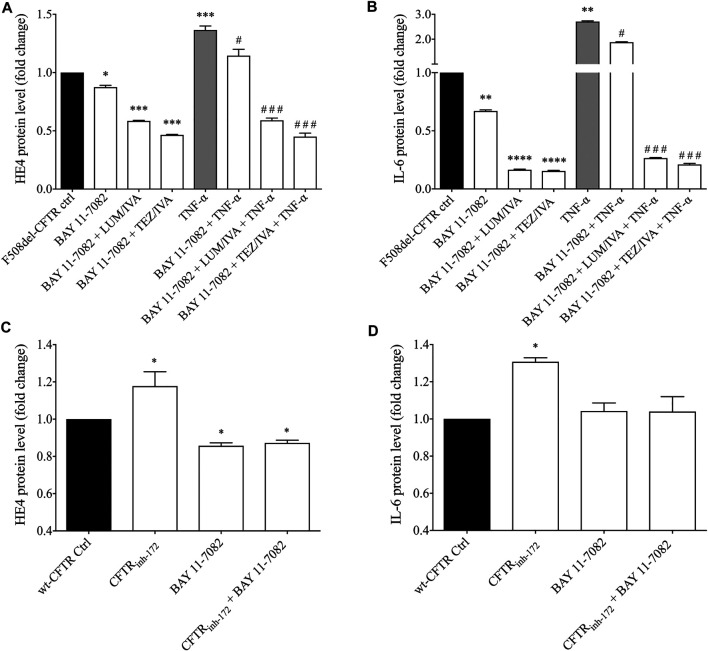
Measurement of HE4 and IL-6 concentrations in non-activated and TNF-α activated F508del-CFTR CFBE 41o^−^ cells in the presence of NF-κB pathway inhibitor and CFTR modulators. BAY 11-7082 (5 μM) was applied to attenuate NF-κB-mediated inflammatory signaling in relation to basal and TNF-α induced HE4 expression in F508del-CFTR CFBE 41o^−^ cells under LUM/IVA or TEZ/IVA or vehicle for 24 h. TNF-α-induced HE4 **(A)** and IL-6 **(B)** expression was prevented by BAY 11–7082, and an even stronger decrease in HE4 and IL-6 was observed when NF-κB inhibitor and CFTR modulators were administered together vs. samples with TNF-α and DMSO. When wt-CFTR CFBE 41o^−^ cells were treated with CFTR_inh172_ with or without BAY 11-7082, CFTR_inh172_ (20 μM) could not elevate HE4 level after incubation with NF-κB inhibitor suggesting the role of NF-κB associated with CFTR **(C)** Changes in IL-6 with the same trend indicates a close relationship between CFTR function and pro-inflammatory conditions in CF **(D)** Mean ± SEM, n = 5–6 samples/condition. ^*^ *p* < 0.05, ^**^ *p* < 0.01, ^***^ *p* < 0.001 and ^****^ *p* < 0.0001 vs. control samples with DMSO; ^#^ *p* < 0.05 and ^###^ *p* < 0.001 vs. control samples with TNF-α and vehicle.

As we consistently detected enhanced levels of HE4 in the presence of abnormal CFTR function in CFBE 41o^−^ cells bearing F508del-CFTR in contrast to wt-CFTR cells under non-activated and TNF-α activated conditions, we raised the question whether increased HE4 expression in CF is under the regulation of NF-κB mediated pathway directly via impaired CFTR function and related pro-inflammatory stimuli. For this purpose, pretreatment with specific NF-κB pathway inhibitor BAY 11-7082 was used in F508del-CFTR CFBE 41o^−^ cells in the absence or presence of CFTR modulators and TNF-α treatment. In these cell culture samples, supernatant HE4 and IL-6 protein levels were determined. We found that BAY 11-7082 mediated inhibition alone significantly lowered baseline HE4 (*p* < 0.05) and IL-6 levels (*p* < 0.01) after 24 h, while BAY 11-7082 with LUM/IVA or TEZ/IVA resulted in a much stronger reduction in the levels of both proteins (*p* < 0.001; *p* < 0.0001, respectively) (Figures 7A,B). TNF-α-induced HE4 expression was also hindered to a large degree via BAY 11-7082-related inhibition of the NF-κB pathway (*p* < 0.05). Moreover, we noted further decrease in HE4 and IL-6 supernatant concentrations when NF-κB pathway inhibitor and CFTR modulators were administered together vs. control samples with TNF-α and DMSO (*p* < 0.001) (Figures 7A,B). In turn, CFBE 41o^−^ cells with wt-CFTR were treated with CFTR_inh172_ with or without BAY 11-7082. In contrast to increased HE4 supernatant concentrations, CFTR_inh172_-based inhibition of CFTR was not associated with elevated HE4 concentrations in the supernatant in the presence of the NF-κB pathway inhibitor (*p* < 0.05) (Figure 7C). Detected changes in IL-6 supernatant concentrations had similar patterns which underscores the close association between CFTR dysfunction and the generally increased pro-inflammatory status in CF (Figure 7D). In summary, HE4 concentrations measured in the supernatants are not only modulated via the NF-κB pathway and pro-inflammatory signaling, but also directly influenced by CFTR in CFBE cell cultures *in vitro*.

## Discussion

In this study, we have provided evidence that enhanced *in vitro* expression of HE4 is regulated by CFTR in CFBE cells. Our results add to the increasing line of evidence that CFTR is not only one of the critical regulators of epithelial fluid balance across various apical epithelial membranes, but may also modulate inflammatory signaling (Eidelman et al., 2001). We have recently reported high concentration of HE4 in serum samples accompanied with its elevated expression in airway epithelial biopsy specimens of CF individuals (Nagy et al., 2016). In addition, HE4 level was around two-fold higher in the supernatant of F508del-CFTR vs. wt-CFTR CFBE cells (Nagy et al., 2016). HE4 or WFDC2 protein belongs to the whey acidic protein four-disulfide-core (WFDC) protein family, and its members, such as Secretory Leukocyte Protease Inhibitor (SLPI) and Elafin (PI3) possess anti-protease, anti-bacterial and anti-inflammatory properties (Small et al., 2017). Due to their abundance in the lung, they have been proposed to be involved in lung homeostasis and protection of the lung from proteolytic “attacks” (Bingle et al., 2006; Small et al., 2017). On the other hand, the mechanism of increased production of HE4 in CF airway epithelial cells is yet unknown.

To date, it is still unclear whether CFTR modulators influence airway inflammatory response via moderating abnormal innate immunity or by epithelial cell activation (Hisert et al., 2017). Latest *in vitro* investigations described downregulated IL-1β and IL-18 levels derived from lipopolysaccharide (LPS)-stimulated monocytes after LUM/IVA or TEZ/IVA treatment (Jarosz-Griffith et al., 2020), reduced secretion of pro-inflammatory cytokines with restored macrophage function in response to *P. aeruginosa* after single IVA or combined LUM/IVA therapy (Barnaby et al., 2018), and depressed CXCL8 expression and p38 MAPK phosphorylation in CFBE cells in response to Orkambi^®^ treatment (Ruffin et al., 2018). Accordingly, CFTR corrector and potentiator drugs may have anti-inflammatory capacities through rescued CFTR function and reduced sweat chloride concentration (Ramsey et al., 2011; Hisert et al., 2017). Of note, this is only indirect evidence from clinical trials and post-marketing studies that has not been duly validated *in vitro*. Moreover, these drugs were not always clinically effective in terms of sustained decrease of chronic bronchial inflammation based on studied biomarkers in the sputum (Rowe et al., 2014), and their anti-inflammatory properties could even gradually vanish after 36 h of treatment (Jarosz-Griffith et al., 2020). In this regard, previous studies reported that the general level of inflammation is increased via constitutive activation of the NF-κB pathway in CF due to CFTR dysfunction that drives higher production of pro-inflammatory cytokines (e.g. IL-8) even in the absence of pathognomonic infectious agents (Vij et al., 2009; Hunter et al., 2010; Wang et al., 2016). Recent clinical studies provide evidence that administration of CFTR modulators normalizes sweat chloride concentration together with a substantial correction of CFTR function in majority of treated subjects (Ramsey et al., 2011; Wainwright et al., 2015; Taylor-Cousar et al., 2017).

Therefore, in this study, we aimed to comprehensively analyze how in our opinion promising biomarker HE4 could be influenced by the administration of CFTR modulators, since the pathogenesis of upregulated HE4 expression in CF is not fully understood. Furthermore, we aimed to provide evidence how treatment with CFTR correctors and potentiators could be monitored using HE4 as a plasma biomarker in routine clinical practice and what the particular diagnostic value of HE4 is in monitoring degree of decreased inflammation in CF patients due to restored function of CFTR. Consequently, we investigated for the first time whether abnormal HE4 expression is directly linked to CFTR dysfunction and is associated with NF-κB pathway in CF. We took aforementioned clinical and laboratory evidence into account when designing our methodological approaches.

For this purpose, we analyzed the change in basal and TNF-α-induced HE4 levels when CFTR function and NF-κB signaling were pharmacologically modulated in CFBE 41o^−^ cells cultures expressing F508del-CFTR in comparison to cells with wt-CFTR. First, there was about 1.5-fold higher basal HE4 concentration in F508del-CFTR vs. wt-CFTR CFBE 41o^−^ cells. Both LUM/IVA and TEZ/IVA significantly lowered HE4 in these cells with F508del-CFTR compared to control samples with vehicle. These compounds were formerly tested for the correction of CFTR in CF epithelial cell cultures via the measurement of CFTR activity *in vitro* (Pranke et al., 2017; Kmit et al., 2019). Here, the rescue of CFTR function by CFTR modulators was observed with whole-cell patch-clamp technique similar to a former publication (Boinot et al., 2014). Based on our data, we considered F508del-CFTR function partially rescued, however, Boinot *et al.* suggested that if the current density was greater than 4 pA/pF after treatment with CFTR modulator, the CFTR activity was corrected (Boinot et al., 2014). As a consequence, HE4 was significantly reduced by both combinations of CFTR modulators. Importantly, TEZ/IVA caused a stronger alteration in both Cl^−^ current and HE4 concentrations compared to LUM/IVA.

Treatment with IVA previously lowered plasma HE4 levels in CF patients bearing a specific Class 3 CFTR pathogenic variant (Nagy et al., 2019), but no data were available on the impact of combined therapy *in vivo* with various CFTR correctors and modulators on plasma HE4 as yet. Hence, we determined plasma HE4 levels in 10 CF individuals homozygous for *p. Phe508del-CFTR* mutation who were taking LUM/IVA regimen. In accordance with *in vitro* data, we provided evidence that this combination of CFTR modulators (Orkambi^®^) led to significantly decreased HE4 plasma concentrations, regardless of individual baseline HE4 concentrations and already after 1 month since the initiation of treatment. Subsequently, we used pharmacological inhibition of CFTR by CFTR_inh172_ in CFBE 41o^−^ cells with wt-CFTR that caused elevated HE4 levels *in vitro*. In contrast, there was a significant decrease in HE4 concentrations after CFTR activator FSK/IBMX treatment in wt-CFTR cells compared to controls. These data are in accordance with previous reports on elevated NF-κB activity with higher IL-8 level following CFTR_inh172_ administration and decreased NF-κB activity after FSK/IBMX treatment in another cell culture (Hunter et al., 2010). In addition, FSK/IBMX treatment resulted in a moderate but statistically significant reduction of HE4 level due to the activation of residual CFTR function of bronchial epithelial cells with F508del-CFTR. However, this particular effect was lower than what we observed in wt-CFTR cells. Finally, when FSK/IBMX was used in combination with CFTR modulators in F508del-CFTR CFBE 41o^−^ cells, HE4 further decreased, especially with TEZ/IVA vs. control samples with DMSO. All these HE4 results are consistent with the change in Cl^−^ current under different treatments (Figure 1A). Based on our results, impaired CFTR function increases baseline HE4 expression in CF airway epithelial cells, which in turn could be efficiently lowered by CFTR modulators.

Next, we wanted to study if *WFDC2/HE4* expression could be upregulated in F508del-CFTR CFBE 41o^−^ cells cultures, so these cells were stimulated *in vitro* with TNF-α for short and long time periods (i.e., for 1 up to 168 h). HE4 mRNA level increased following just 1 h of treatment and remained high for up to 4 h but returned to the baseline by 24 h. Afterward, when HE4 expression was determined up to 168 h, an even higher expression of HE4 was observed in TNF-α stimulated CFBE 41o^−^ cells. To monitor the pro-inflammatory effect of TNF-α on these CFBE cells upon HE4 overexpression, IL6, IL8, and IL1B mRNAs were also analyzed by RT-qPCR in a similar manner. Comparable patterns were found in IL-6 and IL-8 levels as they were upregulated and changed throughout the experiment, while elevated IL1B expression varied only moderately at the different time points. These results indicate that not only pro-inflammatory cytokines are upregulated in CF (Nixon et al., 1998; Cantin et al., 2015), but the expression of HE4 can also be triggered under inflammatory circumstances. As a result, HE4 protein level was significantly elevated after 4 h and gradually increased up to 1 week of treatment, while IL-6 protein concentration was significantly augmented at all time points. Our results are thus reminiscent of a previous report on similar time-dependent regulation of HE4 expression via TLR2 mediated pathway including NF-κB signaling in cancer cells (Janeckova et al., 2015). Similarly, SLPI and Elafin were also induced in response to TNF-α in human alveolar epithelial cells (Sallenave et al., 1994).

Chronic inflammation via upregulated NF-κB signaling leads to gradual progress of structural damage of airways in CF (Cantin et al., 2015). Based on recent findings, ionic imbalance also generated NLRP3-inflammasome activation in CFBE cells exaggerated by LPS/ATP that caused increased IL-18 secretion (Lara-Reyna et al., 2020). To investigate the correlation between enhanced NF-κB pathway caused by CFTR dysfunction and abnormal HE4 expression in CFBE cells, the activity of NF-κB pathway via p65 nuclear translocation was visualized by fluorescence microscopy in wt-CFTR and F508del-CFTR CFBE 41o^−^ cells with or without TNF-α treatment. There was a significantly higher basal level of p65 nuclear positivity in F508del-CFTR vs. wt-CFTR CFBE 41o^−^ cells that was in accordance with former results (Wang et al., 2016). These authors also reported that wt-CFTR regulated TNF-α signaling by enhancing TRADD degradation. By reducing the levels of TRADD, wt-CFTR suppressed downstream the pro-inflammatory NF-κB signaling, while suppression of NF-κB activation failed in CF cells expressing F508del-CFTR (Wang et al., 2016). When our CFBE 41o^−^ cells cultures were activated with TNF-α, the difference in p65 nuclear translocation between normal and deficient CFBE cells was more pronounced. These findings imply that enhanced baseline and triggered NF-κB signaling and abnormal HE4 expression *in vitro* could be related to each other. Therefore, we raised the question of whether application of LUM/IVA or TEZ/IVA treatment could reduce basal and TNF-α induced NF-κB signaling that is responsible for lower HE4 concentrations in the supernatant. We found that CFTR modulators not only attenuated p65 positivity in unstimulated F508del-CFTR CFBE 41o^−^ cells, but a significant reduction was also observed in TNF-α after treatment with CFTR modulators. Decreased baseline and induced IL-6 protein levels in the supernatants of these cells suggest that there are additional anti-inflammatory properties of CFTR modulators apart from decreasing HE4 expression as recently evidenced by others (Ruffin et al., 2018). Overall, these data indicate that the inflammatory status of CF airway epithelial cells could be positively mitigated by CFTR modulators. Our observation is supported by data regarding reduced NLRP3-inflammasome activation in PBMCs observed in CF subjects receiving LUM/IVA or TEZ/IVA via decreased Caspase-1 activity by 3 months of treatment (Jarosz-Griffiths et al., 2020) and by the facts that treatment with Orkambi^®^ restored CFTR dependent chloride efflux (Favia et al., 2020), decreased IL-18 and TNF-α expression in PBMCs (Jarosz-Griffiths et al., 2020), and improved airway epithelial repair (Adam et al., 2018), while Symdeko^®^ downregulated serum IL-1β level in CF subjects (Jarosz-Griffiths et al., 2020). Intriguingly, there were available data, which are contradictory to our results on reduced baseline and TNF-α stimulated IL-6 concentrations in response to LUM/IVA or TEZ/IVA in F508del-CFTR CFBE 41o^−^ cells. Stanton *et al.* reported that PAO1 (MIM:615,854) alone induced a substantial expression of IL-6 and IL-8, while stimulated F508del-CFTR Cl^−^ secretion was reduced despite co-treatment with LUM or LUM/IVA, and IL-6 and IL-8 levels remained unaffected (Stanton et al., 2015). Recently, Laselva *et al.* demonstrated that the levels of IL-6, IL-8 and TNF-α were reduced following Orkambi^®^ and rescued F508del-CFTR HBE cells, which were exposed to PAO1, but only in the presence of antimicrobial peptide or tobramycin (Laselva et al., 2020). In both previous studies, PAO1 resulted in a significant decrease in CFTR expression, but Orkambi^®^ administration was not able to “compensate” for the pro-inflammatory effect of PAO1, which could reflect differences between PAO1 and TNF-α pathway. In our experiments, TNF-α as a pro-inflammatory mediator enhanced IL-6 levels via the NF-κB similar to impaired CFTR function that was prevented by the preincubation with CFTR modulators (Figure 5C) with or without inhibitor BAY 11 7082 (Figure 7B). Based on the immunofluorescence analysis of CFBE 41o^−^ cells for p65 positivity, either LUM/IVA or TEZ/IVA reduced p65 nuclear translocation leading to lower IL-6 and HE4 levels. Thus, despite this discrepancy, we are convinced that our *in vitro* data support the anti-inflammatory properties of CFTR modulators.

Apart from baseline levels of HE4, TNF-α induced HE4 levels also declined with applied CFTR modulators in F508del-CFTR CFBE 41o^−^ cells. Furthermore, TNF-α stimulated CFBE 41o^−^ cells with wt-CFTR demonstrated lowered HE4 levels in the presence of CFTR activator FSK/IBMX. Since we found enhanced level of NF-κB pathway activity in CFBE cells bearing the F508del-CFTR in contrast to wt-CFTR cells at both basal level and upon TNF-α activation, we finally investigated whether HE4 expression could be altered using specific NF-κB pathway inhibitors. For this purpose, the commercially available BAY 11-7082 inhibitor was applied *in vitro* to attenuate NF-κB mediated signaling in deficient CFBE cells in the absence or presence of TNF-α pretreatment and CFTR modulators. Under these experimental conditions we then determined supernatant HE4 and IL-6 protein levels. We found that both inhibitors significantly lowered the baseline and induced protein level of HE4 and IL-6 (Figures 7A,B). Our data implicate that CFTR mediated NF-κB pathway is involved in the regulation of HE4 expression in CFBE cells. The rescue of CFTR function in either unstimulated or TNF-α-activated F508del-CFTR CFBE 41o^−^ cells consistently documented reduced HE4 levels not only in the current *in vitro* samples (Figures 2A, 6A), but also in CF patients who were on CFTR modulator therapy (Figure 2C). These findings were supported with reduced HE4 supernatant levels following BAY 11-7082 CFBE cell culture treatment within the same conditions. In addition, we previously found that in those CF subjects who suffered from more severe inflammation (with acute exacerbation) showing higher CRP values, serum HE4 concentrations were also much higher (Nagy et al., 2016).

Overall, we propose that dual regulation by CFTR and pro-inflammatory signaling via the NF-κB pathway upregulates HE4 expression in CFBE. Bitam and her colleagues formerly reported significantly enhanced CFTR maturation and related chloride currents in F508del-CFTR transfected HeLa cells and primary bronchial epithelial cell cultures after 6 h of treatment with TNF-α, however, CFTR function was only slightly improved within the period of 24 h (Bitam et al., 2015). Therefore, even if TNF-α might rescue CFTR to a certain extent by 24 h under our conditions, HE4 expression was not sufficiently influenced. Earlier, cytokine-mediated induction of Elafin in pulmonary epithelial cells was described to be regulated via an NF-κB site within the proximal promoter of Elafin (Bingle et al., 2001). In addition, a potential NF-κB binding site can be indicated at position -322 relative to the HE4 promoter region (Chen et al., 1998). Hence, we suppose that HE4 is also transcriptionally regulated by the NF-κB, however, further e.g. ChIP-seq-based expression studies are required to support the assertion of any functional relationship.

The manuscript has limitations. Firstly, we have not analyzed whether the absence of CFTR function or the presence of misfolded F508del-CFTR was the cause of increased HE4 expression. Secondly, CF patients on TEZ/IVA medication could not be enrolled for plasma HE4 measurement into this study, because such samples are currently not available, either from CFFT nor from our national studies. Thirdly, the mechanistic relationship between the CF-related inflammation and the expression of HE4 was not fully investigated, thus additional studies are required to reveal further related pathogenetic aspects.

**FIGURE 8:**
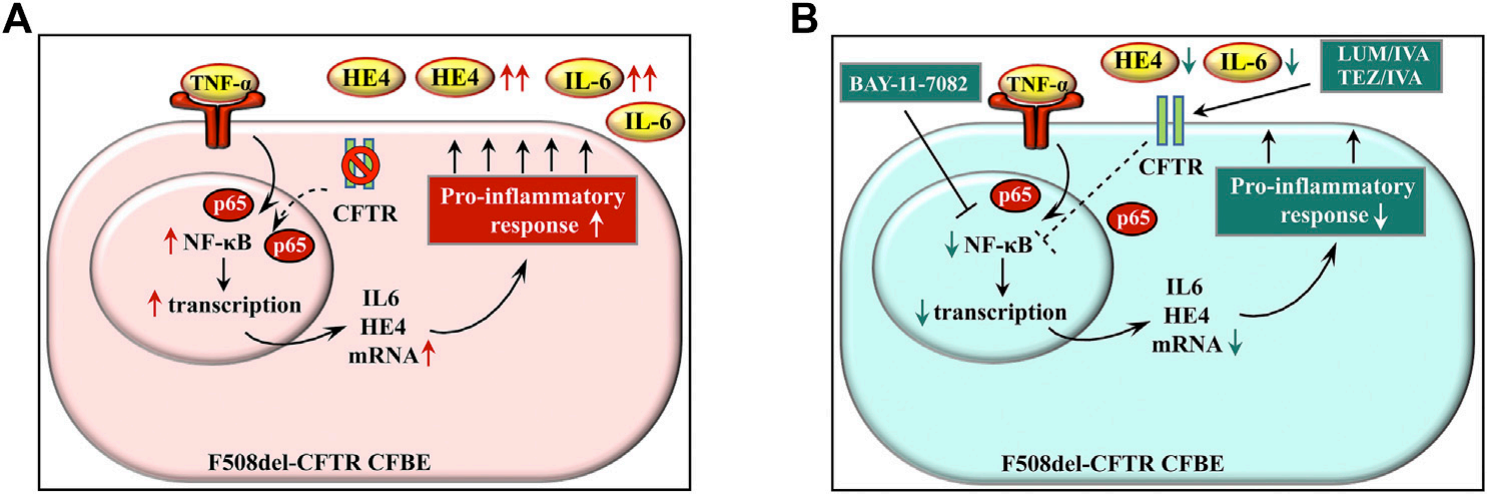
Schematic figure about the model on the regulation of HE4 expression in CF via CFTR and pro-inflammatory signaling. There is an increased basal expression of HE4 with cytokines (e.g., IL-6) in lung epithelial cells of CF that is caused by CFTR dysfunction and external pro-inflammatory stimuli, e.g., TNF-α via the NF-κB pathway **(A)** Blocking the effect of TNF-α by BAY 11-7082 or using CFTR modulators (LUM/IVA or TEZ/IVA) can effectively reduce pro-inflammatory response in CFBE cells by suppressing the NF-κB pathway with a still unknown mechanism (dotted line) that prevents IL-6 and HE4 overexpression **(B)** Abbreviations: CFBE cell: cystic fibrosis bronchial epithelial cell, CFTR: cystic fibrosis transmembrane conductance regulator, TNF-α: tumor necrosis factor alpha, HE4: human epididymis protein 4, IL-6: interleukin-6, NF-κB: nuclear factor-kappa B.

In conclusion, we provide evidence that there is a direct relationship between CFTR function and pro-inflammatory response in F508del-CFTR CFBE cells in terms of increased expression of HE4 measured by its concentrations in the cell culture supernatant *in vitro* (Figure 8). CFTR corrector and potentiator administration restores inflammatory balance via correcting CFTR function and reducing NF-κB p65 nuclear translocation in CFBE cells as reflected by lower HE4 concentrations *in vitro* and *in vivo*. Finally, we believe that our study opens novel research avenues in terms of providing evidence for the diagnostic utility of plasma biomarker HE4 in terms of its use in efficient monitoring of anti-inflammatory properties of CFTR modulators.

## Data Availability

The raw data supporting the conclusions of this article will be made available by the authors, without undue reservation, to any qualified researcher.

## Abbreviations

CF, cystic fibrosis; CFBE, cystic fibrosis bronchial epithelial cell; CFFT, Cystic Fibrosis Foundation Therapeutics; CFTR, CF transmembrane conductance regulator; FEV_1_, forced expiratory volume in 1 second; HE4, human epididymis protein 4; IL-6, interleukin-6; LPS, lipopolysaccharide; NF-κB, nuclear factor-kappa B; PBMCs, peripheral blood mononuclear cells; PEx, pulmonary exacerbation; RT, room temperature; SLPI, Secretory Leukocyte Protease Inhibitor; TNF-α, tumor necrosis factor α; WFDC, whey acidic protein four-disulfide-core; wt-CFTR, wild-type CFTR.
