# Supplementary figures and images for: Enhanced Expression of Human Epididymis Protein 4 (HE4) Reflecting Pro-Inflammatory Status Is Regulated by CFTR in Cystic Fibrosis Bronchial Epithelial Cells
Source: Front Pharmacol. 2021 May 14;12:592184. doi: 10.3389/fphar.2021.592184 (PMC8160512; doi:10.3389/fphar.2021.592184)

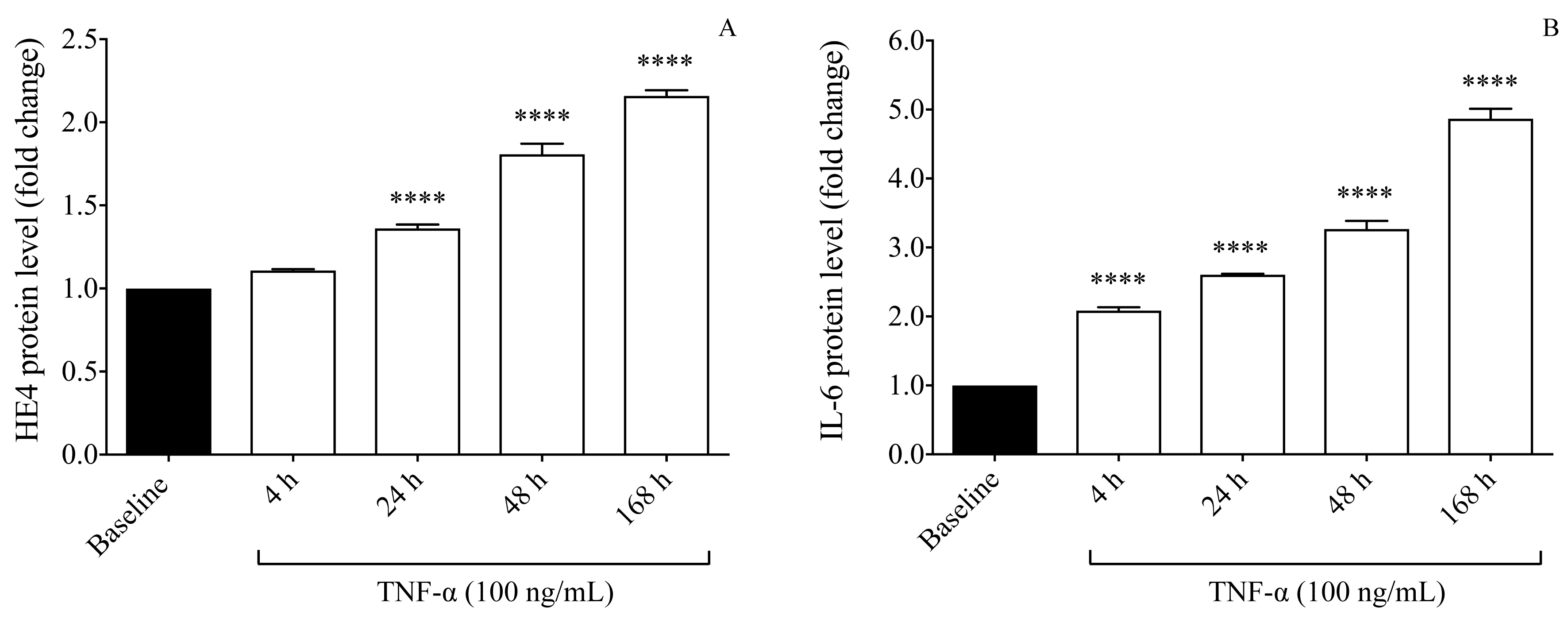

Supplement: Supplementary file 1 [file Image1.TIF]
