# Supplementary material for: Enhanced Expression of Human Epididymis Protein 4 (HE4) Reflecting Pro-Inflammatory Status Is Regulated by CFTR in Cystic Fibrosis Bronchial Epithelial Cells
Source: Front Pharmacol. 2021 May 14;12:592184. doi: 10.3389/fphar.2021.592184 (PMC8160512; doi:10.3389/fphar.2021.592184)
Supplement: Supplementary file 2 [file DataSheet1.docx]

| **Messenger RNAs** | **Forward primers for RT-qPCR** | **Reverse primers for RT-qPCR** |
| --- | --- | --- |
| **HE4** | 5' - ATAGCACCATGCCTGCTTGT - 3' | 5' - TGCTCCTGTGCCTGAGACTA - 3' |
| **IL6** | 5' - GATGAGTACAAAAGTCCTGATCCA - 3' | 5' - CTGCAGCCACTGGTTCTGT - 3' |
| **IL8** | 5' - AGACAGCAGAGCACACAAGC - 3' | 5' - ATGGTTCCTTCCGGTGGT - 3' |
| **IL1B** | 5' - AGCCAGGACAGTCAGCTCTC - 3' | 5' - AGAGGCCTGGCTCAACAA - 3' |
| **RPLP0 (36B4)** | 5' - ATGCAGCAGATCCGCATGT- 3' | 5' - TCATGGTGTTCTTGCCCATCA - 3' |

| **Patient N^o^** | **Age (years)** | **Gender** | **Baseline FEV_1_ (%)** | **Baseline sweat Cl^-^ (mEq/L)** | **Δ sweat Cl^-^ (1 month)** | **Baseline BMI (kg/m^2^)** |
| --- | --- | --- | --- | --- | --- | --- |
| 1 | 24.5 | female | 49.5 | 105 | -14.5 | 20.5 |
| 2 | 25.5 | female | 63.2 | 88 | -44.5 | 25.3 |
| 3 | 14.8 | female | 49.8 | 102 | -23.5 | 21.0 |
| 4 | 12.9 | female | 73.8 | 103 | -19 | 16.6 |
| 5 | 14.5 | male | 93.9 | 106.5 | -13 | 25.1 |
| 6 | 12.1 | male | 94.6 | 93 | -19 | 18.2 |
| 7 | 12.8 | male | 91.1 | 108 | -7.5 | 17.7 |
| 8 | 14.3 | male | 79.4 | 85.5 | -15.5 | 18.5 |
| 9 | 14.1 | male | 71.3 | 108 | -30 | 14.1 |
| 10 | 15.2 | female | 80.1 | 112.5 | -9.5 | 21.3 |

**Suppl. Table 1.**

**Suppl. Table 2.**
